# SmpB and tmRNA Orchestrate Purine Pathway for the Trimethoprim Resistance in *Aeromonas veronii*

**DOI:** 10.3389/fcimb.2020.00239

**Published:** 2020-05-25

**Authors:** Dan Wang, Hong Li, Wasi Ullah Khan, Xiang Ma, Hongqian Tang, Yanqiong Tang, Dongyi Huang, Zhu Liu

**Affiliations:** ^1^Key Laboratory of Tropical Biological Resources of Ministry of Education, School of Life and Pharmaceutical Science, Hainan University, Haikou, China; ^2^Key Laboratory for Sustainable Utilization of Tropical Bioresource, College of Tropical Crops Hainan University, Haikou, China

**Keywords:** *Aeromonas veronii*, trimethoprim, SmpB, tmRNA, purine metabolism

## Abstract

Small protein B(SmpB) cooperates with transfer-messenger RNA (tmRNA) for *trans*-translation to ensure the quality control of protein synthesis in prokaryotes. Furthermore, they regulate cell metabolism separately. According to research, SmpB functions as a transcription factor, and tmRNA acts as a small RNA. Purine pathway has been reported to be related to trimethoprim resistance, including hypoxanthine synthesis, adenosine metabolism and guanosine metabolism. Another reason of drug tolerance is the efflux pump of the bacterium. In transcriptomic data, it was shown that the expression of some related enzymes in adenosine metabolism were raised significantly in *smpB* deletion strain than that of wild type, which led to the differential trimethoprim resistance of *Aeromonas veronii* (*A. veronii)*. Furthermore, the metabolic products of adenosine AMP, cAMP, and deoxyadenosine were accumulated significantly. However, the expressions of the enzymes related to hypoxanthine synthesis and guanosine metabolism were elevated significantly in *ssrA (small stable RNA, tmRNA) deletion strain*, which eventually caused an augmented metabolic product xanthine. In addition, the deletion of *ssrA* also affected the significant downregulations of efflux pump *acrA/acrB*. The minimal inhibitory concentrations (MIC) were overall decreased after the trimethoprim treatment to the wild type, Δ*smpB* and Δ*ssrA*. And the difference in sensitivity between Δ*smpB* and Δ*ssrA* was evident. The MIC of Δ*smpB* was descended significantly than those of wild type and Δ*ssrA* in M9 medium supplemented with 1 mM adenosine, illustrating that the adenosine metabolism pathway was principally influenced by SmpB. Likewise, the strain Δ*ssrA* conferred more sensitivity than wild type and Δ*smpB* in M9 medium supplemented with 1mM guanosine. By overexpressing *acrA*/*acrB*, the tolerance to trimethoprim was partially recovered in Δ*ssrA*. These results revealed that SmpB and tmRNA acted on different branches in purine metabolism, conferring the diverse trimethoprim resistance to *A. veronii*. This study suggests that the *trans*-translation system might be an effective target in clinical treatment of *A. veronii* and other multi-antibiotic resistance bacteria with trimethoprim.

## Introduction

*Aeromonas veronii* (*A. veronii*) is a pathogen of aquatic animals and humans. It is widely found in freshwater and seawater with a strong pathogenic ability, causing fish skin ulceration, visceral hemorrhage, ascites, and other symptoms (Liu et al., 2016). Clinical studies have reported that *A. veronii* can also cause human gastroenteritis, endocarditis, bacteremia and other diseases (Aguilera-Arreola et al., 2007; Chuang et al., 2011). *A. veronii* is resistant to spectinomycin, gentamicin, streptomycin, and kanamycin (Zhang et al., 2019). Bacterial drug resistance makes it more difficult to control pathogens (Leal et al., 2019; El Mekes et al., 2020). Studies have shown that *trans*-translation system composed of SmpB (Small protein B) and tmRNA (transfer-messenger RNA) can release ribosomal block caused by translation errors, thus ensuring ribosome efficiency and protein synthesis (Keiler, 2015), which has an important impact on bacterial drug resistance (Venkataraman et al., 2014; Sharkey et al., 2016).

In addition to the *trans*-translation function of SmpB and tmRNA, they also play other roles in bacteria. As a transcription factor, SmpB participates in transcriptional regulation of *bvgS* and sRNA of *A. veronii* (Liu et al., 2015; Wang et al., 2019). SmpB also affects the transcription of sRNA and the tolerance to environmental stress (Wang et al., 2019). Analogously, tmRNA can act as sRNA and interact with *crtMN* mRNA to affect the synthesis of *Staphylococcus aureus* pigment (Liu et al., 2010). Results of the present study show that a double knockout of *smpB* and *ssrA (tmRNA, small stable RNA)* makes *A. veronii* more sensitive to trimethoprim.

It is reported that bacteria could develop acquired drug tolerance when treated with Trimethoprim (Ma et al., 2018). Trimethoprim affects the synthesis of nucleotides and purine metabolism of *A. veronii* by inhibiting dihydrofolate reductase (Sangurdekar et al., 2011). Purine metabolism also affects the bacterial tolerance to trimethoprim (Sah et al., 2015; Stepanek et al., 2016). This study explored the molecular mechanism of SmpB and tmRNA in the tolerance of *A. veronii* to trimethoprim, mainly through purine metabolism. In Δ*smpB*, seven adenine related enzyme expression upregulated, and the content of adenosine increased. In Δ*ssrA*, a large number of guanine related enzymes upregulated, the content guanine and hypoxanthine contents increased. SmpB and tmRNA affect two branches of purine metabolism, adenine synthesis and guanine synthesis, respectively. The sensitivity of *A. veronii* to trimethoprim increased with the addition of exogenous purine products. More different, Δ*ssrA* efflux pump related gene expression was decreased, and the overexpression of *acrAB* made Δ*ssrA* increased the resistance of trimethoprim. This study explained the reason for decreased MIC of trimethoprim, and the different influence between Δ*smpB* and Δ*ssrA* on purine pathway was also found.

## Methods

### Strains

The strain information was listed in Table 1. *A. veronii* was isolated from the diseased tissues of grass carp (Liu et al., 2015) and cultured with M9 Minimal Medium (M9) at 30°C, 150 r/min, and 50 ug/mL ampicillin. *SmpB* knockout strain and *ssrA* knockout strain was derived from the preserved strain in this laboratory (Liu et al., 2015). The *smpB* and *ssrA* double knockout strain (Δ*smpB*_*ssrA*) and *ssrA* mutant complemented with *acrAB* sequence (Δ*ssrA::acrAB*) were constructed in this study. *E coli* WM 3064 assisted the vector introduction into *A. veronii* for the construction of overexpressed strains and knockout strains. *E coli* WM3064 was Diaminopimelic acid (DAP) dependent and cultured in LB medium at 37°C with DAP concentration of 0.3mM. All of the medium composition belongs to inorganic salts. For the 5× stock: 64 g Na_2_HPO_4_, 15 g KH_2_PO_4_, 5 g NH_4_Cl, 2.5 g NaCl, and 1 liter of high-quality distilled water. Mix 1 ml of 1 M MgSO_4_.7H_2_O, 10 ml of 20% glucose, 100ul of 1M CaCl_2_ and 200mL of 5× stock solution and adjust to 1000ml with distilled H_2_O.

**Table 1:** Strains and plasmids used in this paper.

**Strains or plasmids**	**Traits**	**Sources**
*E. coli* WM3064	Gene cloning strain	This lab
*Aeromonas veronii*	Wild type strain	This lab
*smpB_ssrA* mutant (Δ*smpB_ ssrA)*	*smpB* and *ssrA* deletion mutant	This paper
Δ*smpB_ ssrA*:: c	*A complementary strain of smpB* and *ssrA* deletion mutant	This paper
*smpB* mutant	*smpB* deletion mutant	This lab
Δ*smpB:: smpB*	*A complementary strain of smpB* deletion mutant	This lab
*ssrA* mutant	*ssrA* deletion mutant	This lab
Δ*ssrA:: ssrA*	*The complementary strain of ssrA* deletion mutant	This lab
*ssrA* mutant *with acrAB*	Over-expressing *acrAB* in *ssrA* mutant strain	This paper
pre112	Gene cloning vector	This lab
pBBR1MCS-2	Gene cloning vector	This lab
pBBR *acrAB*	*acrAB* overexpression vector	This paper

### Plasmids

The construction vectors and primers used in this study are listed in Tables 1, 2. Suicide plasmid pRE112 was used to construct knockout vectors. And the assembly knockout vectors of *smpB_ssrA* sequence were constructed by double enzyme digestion and specific connection and were integrated with the genome under 6% sucrose to complete homologous recombination (Liu et al., 2015). Plasmid pBBR1MCS-2 was used to construct a vector that overexpressed efflux pump-related gene *acrAB* in *A. veronii*. DNA sequence *acrA* and *acrB* were introduced into the pBBR1MCs-2 plasmid, as well as the upstream and downstream primers needed for vector construction respectively. The plasmid was transferred into Δ*ssrA* strain through affinity experiment to construct the overexpressed strain. The overexpressed strains were screened using plates containing both Kanamycin and Ampicillin resistance and verified by sequencing.

**Table 2:** Primers and plasmids used in this paper.

**Names**	**Sequences (5^**′**^-3^**′**^)**	**Usage**
WP_041202667.1-F	ATGGTCGCAGAGCTTGTC	Strain validation
WP_041202667.1-R	CAGCACAATAGAACACCAGAC	Strain validation
*acrA Sal* I F	ACGCGTCGACTTGGTATCG GCTGGGGATTG	*acrAB* vector construction
*acrB EcoR* I R	CCGGAATTCATGAGCGTCGGGAGAG	*acrAB* vector construction
pBBR1MCS-2 F	GGCACCCCAGGCTTTACACT	Complement plasmid validation
pBBR1MCS-2 R	GATGTGCTGCAAGGCGATTAAG	Complement plasmid validation
pre112 F	GTGCGTACCGGGTTGAGAAG	pre112 validation
pre112 R	CGCCCTTAAACGCCTGGTTG	pre112 validation
Iden-F	GGTCAGACACCGTATACCTC	Deleted sequence validation
Iden-R	TCAAGAGATCGCCATGAGTC	Deleted sequence validation
Up-*smpB* F	CGGGGTACCGAGATCAAACTCCACCTTGCAG	Upstream arm of *smpB* amplification
Up-*smpB* R	CCGGAATTCGACGGGCGATTTCCGGCAAT	Upstream arm of *smpB* amplification
Down- *ssrA* F	CCGGAATTCGCTCCACCAAACAATGTTCC	Downstream arm of *ssrA* amplification
Down- *ssrA* R	CCCGAGCTCGCGTTAGCTTCTTGTTCTG	Downstream arm of *ssrA* amplification

### Minimum Inhibitory Concentration Test

Micro broth dilution method: Antibiotics were added to the sterile 96-well plates at final concentrations of 64, 32, 16, 8, 4, 2, 1, 0.5, 0.25, and 0.125 ug/mL. Then, 10-E6 CFU broth was added to each well to a final volume of 200 μL. The 96-well plate was sealed with parafilm and cultured at 30°C with shaking at 150 r/min for 24 h. The minimum drug concentration that completely inhibited the growth in the well was considered as the minimum inhibitory concentration. The experiment was repeated 3 times.

### Transcriptomic Analysis

The cells were collected, lysed, and the sample RNA was extracted with phenol-chloroform. The concentration and quality of the RNA samples were tested with the Agilent 2100. DNase I was used to remove double-stranded DNA, and a Ribo-Zero Magnetic Kit was used to remove human RNA. Reverse transcription was performed with random primers and first strand cDNA which was used as a template to synthesize the second strand. The linker sequence was attached to the 3′ end of the cDNA fragment. The cDNA sequence was amplified with a primer cocktail, and the purified product was sequenced on a HiSeq Xten (Illumina, San Diego, CA, USA) platform. The sequencing depth was chain-specific sequencing for 2 Gb of clean data. HISAT was attempted for genome assembly, analysis of potential coding sequence analysis and to identify new transcripts that may be present. Differences in gene transcription levels between wild-type and knockout strains were analyzed by Bowtie 2, and RPKM was used to normalize gene expression levels. The results of gene expression were calculated using the Benjamini-Hochberg false discovery rate (FDR). The differentially expressed transcripts were tested for log-fold change and the *p* value was corrected with FDR <0.001. The results of the differentially expressed genes were analyzed using GO classification, and differential gene expression in the pathway was com*pared* with the entire genomic background using hyper geometric analysis. Value of *p* < 0.05 is considered to be a differential metabolic pathway. The accession number of reference genome is NO. CP012504.1. GEO accession number is GSE120603, and the URL of accession website is https://submit.ncbi.nlm.nih.gov/subs/sra/SUB6133286. The DESeq.2 package in R was applied to estimate the fold changes and to perform other analysis.

### Metabolomics Analysis

The non-target metabolomics and lipidomics detection platform (UHPLC-QTOF-MS) was applied to metabolomics for the detection of *A. veronii* samples. UHPLC-QTOF-MS includes Ultra-Performance Liquid Chromatography 1290UHPLC (Agilent), ACQUITY UPLC BEH Amide column 1.7 um, 2.1^*^100 mm (Waters) and High-Resolution Mass Spectrometry Triple TOF 6600 (AB Sciex). The original mass spectrum was converted to the mzXML format using Proteo Wizard software, and the peaks were identified using the R Programming Language package (Version 3.2) and self-built secondary mass spectrometry data. URL of accession website is: www.ebi.ac.uk/metabolights/MTBLS1411.

### Statistical Analysis

Statistical data were analyzed using the Statistical Package for the Social Science (SPSS) version 20.0 (SPSS, Chicago, IL, USA) and GraphPad Prism version 8.0 (GraphPad, San Diego, CA, USA). The results are presented as the mean values of three independent experiments with standard deviation (SD) using one-way analysis of variance (ANOVA). Values of *p* < 0.05 or 0.01 were represented as significant or extremely significant, respectively.

## Results

1. The trimethoprim tolerance of Δ*smpB_ssrA* to was decreased than wild type *A. veronii*.

**Figure 1:**
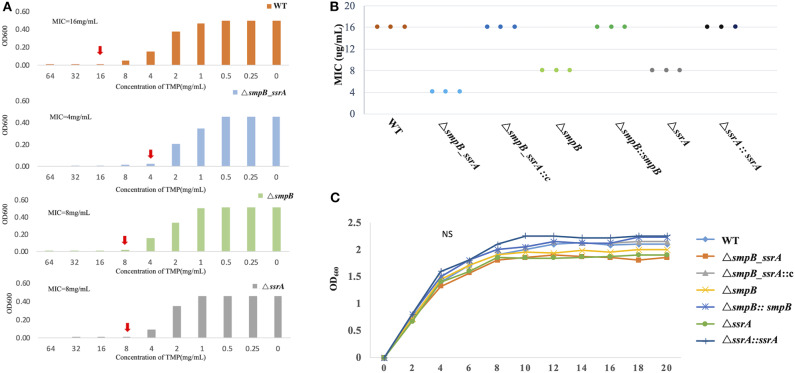
Minimum inhibitory concentration (MIC) value of trimethoprim. **(A)** The red arrow indicates the concentration of trimethoprim in M9 medium that prevented the growth of *Aeromonas veronii*. **(B)** The MIC of the complementary strain of the three mutants. **(C)** The growth curve of wild type *A. veronii*, Δ*smpB*, Δ*ssrA, and* Δ*smpB_ ssrA, and the complementary strains*.

The minimum inhibitory concentration (MIC) was tested by trimethoprim gradient concentration. The trimethoprim MIC of Δ*smpB_ssrA* was 2 ug/mL. Compared with wild type *A. veronii*, the strain of Δ*smpB_ssrA* was more sensitive to trimethoprim (Figure 1A). In order to distinguish the function of SmpB and tmRNA on trimethoprim, Δ*smpB* and Δ*ssrA* strains were used in this study. The MIC data showed that Δ*smpB* and Δ*ssrA* were more sensitive than the wild type, which was still higher than Δ*smpB_ssrA*. The MIC data of complementary strain was tested (Figure 1B). The changes between wild type and knockout strains were rescued by transferring their pBBR plasmids into the deficient strain. The growth curve was tested to prove that the MIC value was not influenced by the non-obvious difference on growth (Figure 1C) This phenomenon suggested that the functions of SmpB and tmRNA were different from *trans*-translation in the presence of trimethoprim.

2. The difference between Δ*smpB* and Δ*ssrA* occurred at the transcriptional level.

**Figure 2:**
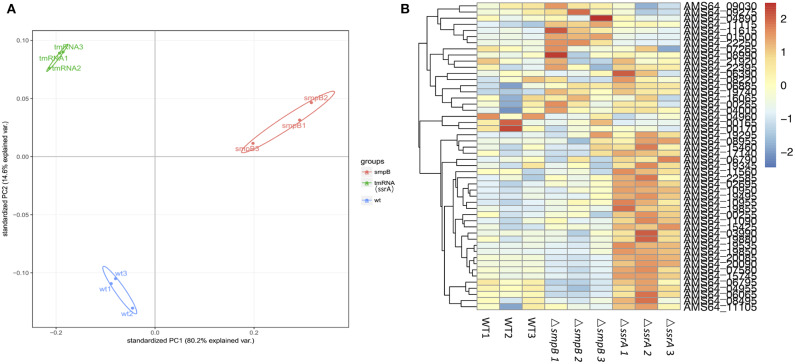
Transcriptomic data of *Aeromonas veronii*. **(A)** Principal component analysis (PCA) diagram of wild type, Δ*smpB* and Δ*ssrA* data. **(B)** Heat map of expression levels of genes involved in purine metabolism and efflux pump synthesis of wild type, Δ*smpB* and Δ*ssrA* strains. There were three samples of wild type, Δ*smpB* and Δ*ssrA* for transcriptome sequencing and analysing. The reads were mapped to reference genome with the accession NO. CP012504.1.

In order to explore the differences on function of SmpB and tmRNA, the transcriptomic sequencing was taken to test the expression of stationary phage Δ*smpB* and Δ*ssrA* stains. The transcriptomic results showed that there was a big difference between Δ*smpB* and Δ*ssrA* (Figure 2A). As the expression of guanosine related enzymes in Δ*ssrA* purine metabolism was significantly higher than that in wild type *A. veronii*. Simultaneously, the Expression of adenosine related enzymes in Δ*smpB* purine metabolism was significantly higher than wild type *A. veronii* (Figure 2B).

3. The content of purine metabolites in Δ*smpB* and Δ*ssrA* were different.

**Figure 3:**
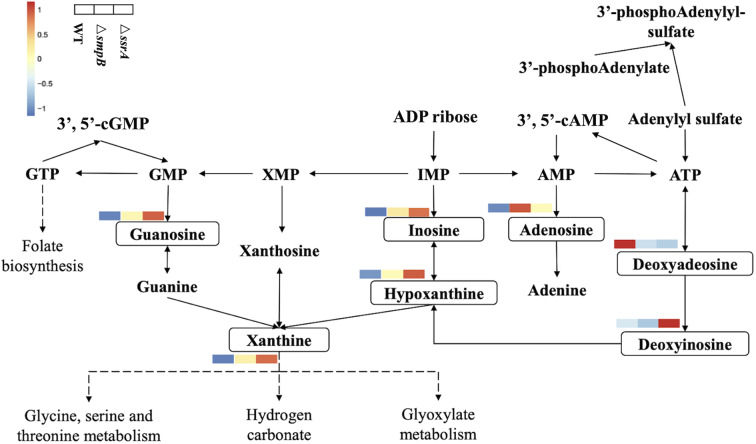
Simplified diagram of *Aeromonas veronii* purine metabolic pathway. The metabolites with a change between wild-type, Δ*smpB* and Δ*ssrA* strains were framed by solid line and presented by heat-map. Compounds with no significant difference in content of the three strains were not labeled.

Metabonomic analysis of Δ*smpB* and Δ*ssrA* strains was done because of significant differences in the transcriptomic data. Metabolomics results showed that guanosine metabolites and adenosine metabolites showed up-regulation in the purine metabolic pathway separately in Δ*smpB* and Δ*ssrA* (Figure 3). The content of adenosine, AMP, cAMP, and deoxyadenosine in Δ*smpB* were increased significantly. Whereas the contents of xanthine and guanosine in Δ*ssrA* were significantly increased.

4. Trimethoprim tolerance of Δ*ssrA* was enhanced by *acrAB* overexpression.

**Figure 4:**
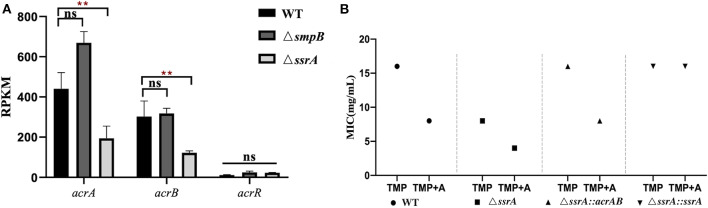
Trimethoprim tolerance of Δ*ssrA* was enhanced by *acrAB* overexpression. **(A)** Expression of efflux pump related gene *acrA* and *acrB* in wild type, Δ*smpB* and Δ*ssrA* strains. **(B)** MIC value of wild type, Δ*ssrA* strains affected by overexpression of *acrAB*. A represents the metabolite substrate 1 mM adenosine. **represents *p* < 0.01, and ns represents no significant difference.

The expression of efflux pump gene *acrA* and *acrB* in Δ*ssrA* was downregulated, but no significant change in Δ*smpB* (Figure 4A). The *acrAB* over-expression vector pBBR_*acrAB*, which was constructed with *acrAB* promoter, were introduced into Δ*ssrA*. Trimethoprim MIC test results showed that the MIC of Δ*ssrA::acrAB* similar to wild type strain (Figure 4B). The tolerance to trimethoprim is enhanced by over-expression of *acrAB* in Δ*ssrA*. Under the condition of 1 mM adenosine in M9, the MIC of Δ*ssrA::acrAB* strain increased to 8 mg/mL, which is lower than the wild type.

5. The trimethoprim tolerance of Δ*smpB* and Δ*ssrA* was weakened by exogenous purine metabolites.

**Figure 5:**
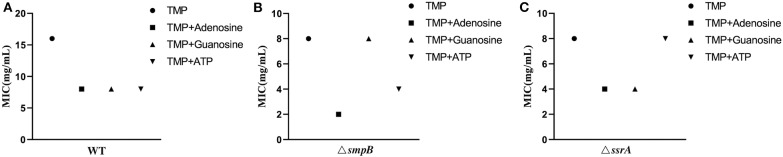
MIC value of trimethoprim under conditions of adenosine, guanosine and adenosine triphosphate. **(A)** MIC value of wild type with 1 mM adenosine, 1 mM guanosine, and 10 mM ATP in M9 medium. **(B)** MIC of Δ*smpB* with 1 mM adenosine, 1 mM guanosine, and 10 mM ATP in M9 medium. **(C)** MIC of Δ*ssrA* with 1 mM adenosine, 1 mM guanosine, and 10 mM ATP in M9 medium.

Excessive purine metabolites in M9 medium resulted in a decrease in MIC value, making the wildtype of *A. veronii* more sensitive to trimethoprim (Figure 5A, Supplementary Table 1). The MIC value of Δ*smpB* was decreased by adding exogenous 1mM adenosine and 10mM ATP but was not changed by excessive guanosine (Figure 5B) and complementary strains (Supplementary Table 1). These results implied that the increase of adenosine in adenosine metabolic pathways could make Δ*smpB* more sensitive to trimethoprim. Adding exogenous adenosine and guanosine, the MIC value of Δ*ssrA* was decreased. Conversely, there was no change between the MIC of Δ*ssrA* and the wild type cultivated by 10 mM ATP in M9 medium (Figure 5C). And 1mM guanosine in M9 medium had an effect on MIC value of Δ*ssrA*. These results demonstrated that the differences in the regulation of purine metabolites led to differences in their tolerance to trimethoprim between Δ*smpB* and Δ*ssrA*.

## Discussion

Trimethoprim inhibits the synthesis of tetrahydrofolic acid, a carbon unit carrier, by affecting the activity of dihydrofolate reductase, thereby affecting the growth and metabolism of bacteria (Bertacine Dias et al., 2018). The products of purine metabolism will be reduced under the inadequate condition of one carbon unit (Sah et al., 2015). However, in the *A. veronii* metabonomic data, the decrease in folate metabolism and the increase in purine pathway-related enzymes were found to occur at the same time as the significant up-regulation of the purine metabolic pathway. The upregulation trend of Δ*ssrA* was more sensitive to trimethoprim than that of Δ*smpB*. The decrease of MIC value of trimethoprim to Δ*smpB* and Δ*ssrA* were not consistent with the double knockout strain. This indicated different functions of SmpB and tmRNA acting on the tolerance of trimethoprim. These results suggested that the reduced tolerance of trimethoprim in the double-knockout strain may be less relevant to the function of the *trans*-translation system.

ATP is known to be the most effective energy to physiological reactions. As an important products of purine metabolism, ATP was predicted to be beneficial to the growth of *A. veronii*. In this study, 10mM ATP led to a decrease in trimethoprim MIC value, suggesting that excessive ATP weakened the tolerance of *A. veronii* to trimethoprim. It was speculated that the heightened need of ATP increased the challenge to the one carbon unit synthesis of the organism. The role of efflux pump had been explored in drug resistance to fluoroquinolones in gram negative bacteria. AcrAB-TolC efflux system, which has a physiologic role of pumping out bile acids and fatty acids to lower their toxicity. The decreased expression of AcrAB also contributed to the accumulation of ATP. Therefore, it is speculated that ATP in a dynamic balance that It showed little influence upon the value of MIC.

Recent studies had been focused on the assistance of SmpB in binding to the stalled ribosome to release it (Keiler, 2015; Buskirk and Green, 2017). In Δ*ssrA*, the expression of genes related to guanine synthesis was significantly upregulated and the mRNA of genes related to effusion pump was significantly decreased, which did not occur in Δ*smpB*. On the contrary, the expression adenine-related enzymes of Δ*smpB* were significantly increased, which were not repeated in Δ*ssrA*. This indicates that SmpB and tmRNA have independent and non-interference functions.

The difference in tolerance between double knock out strain Δ*smpB_ssrA* and single deletion strain Δ*smpB* and Δ*ssrA* indicated the distinctive functions. The transcriptional and metabolites data showed separate regulation of SmpB and tmRNA. The changes of Δ*ssrA* were more significant. The trimethoprim MIC value results proved that the efflux pump AcrAB-TolC was affected by *ssrA* deletion. Therefore, this study demonstrated that SmpB and tmRNA act on different branches of purine metabolism, showing an effect on the tolerance to trimethoprim. A second collaboration between SmpB and tmRNA was completed by regulating purine metabolic pathway.

## Data Availability Statement

The raw data supporting the conclusions of this article will be made available by the authors, without undue reservation, to any qualified researcher.

## Author Contributions

ZL, YT, DH, and DW contributed the conception and design of the study. DW, HL, XM, and HT performed the statistical analysis. DW, WK, and ZL drafted the manuscript. All authors contributed to manuscript revision, read and approved the submitted version.

### Conflict of Interest

The authors declare that the research was conducted in the absence of any commercial or financial relationships that could be construed as a potential conflict of interest.
